# Circ_0078767 suppresses non‐small‐cell lung cancer by protecting RASSF1A expression via sponging miR‐330‐3p

**DOI:** 10.1111/cpr.12548

**Published:** 2018-12-03

**Authors:** Ting Chen, Zuozhang Yang, Chao Liu, Li Wang, Jun Yang, Long Chen, Wenhui Li

**Affiliations:** ^1^ Department of Nuclear Medicine Tumor Hospital of Yunnan Province, The Third Affiliated Hospital of Kunming Medical College Kunming China; ^2^ Department of Orthopedics Tumor Hospital of Yunnan Province, The Third Affiliated Hospital of Kunming Medical College Kunming China; ^3^ Department of Radiotherapy Tumor Hospital of Yunnan Province, The Third Affiliated Hospital of Kunming Medical College Kunming China; ^4^ Department of Radiology Tumor Hospital of Yunnan Province, The Third Affiliated Hospital of Kunming Medical College Kunming China; ^5^ Department of PETCT Tumor Hospital of Yunnan Province, The Third Affiliated Hospital of Kunming Medical College Kunming China

**Keywords:** circ_0078767, miR‐330‐3p, non‐small‐cell lung cancer, qRT‐PCR, RASSF1A

## Abstract

**Objectives:**

This study was designed to investigate the role of circ_0078767/miR‐330‐3p/RASSF1A in non‐small‐cell lung cancer (NSCLC). Bioinformatic analysis was performed to screen for the differentially expressed genes in NSCLC tissues from adjacent lung tissues.

**Materials and Methods:**

qRT‐PCR was used to detect the RNA expression of genes in cells and tissues, and Western blot was conducted to determine the protein levels of RASSF1A in tissues and cells. A miRanda algorithm was used to predict the targeted relationship among RNAs. A dual‐luciferase reporter gene assay was conducted to verify the targeted relationship. Flow cytometry was performed to investigate the effects of circ_0078767/miR‐330‐3p/RASSF1A on cell cycle progression and apoptosis. A CCK‐8 assay was conducted to explore the effects of circ_0078767/miR‐330‐3p/RASSF1A on cell proliferation. A transwell invasion assay was completed to study the effects of circ_0078767/miR‐330‐3p/RASSF1A on cell invasion. Lastly, an in vivo assay was conducted to investigate the effects of circ_0078767/miR‐330‐3p/RASSF1A on tumour development.

**Results:**

Circ_0078767 and RASSF1A were downregulated, while miR‐330‐3p was upregulated in NSCLC tissues than that in adjacent tissues. miR‐330‐3p had a binding relationship with circ_0078767 and RASSF1A. The overexpression of circ_0078767 and RASSF1A or the underexpression of miR‐330‐3p significantly suppressed NSCLC cell viability, cell cycle progression and invasion while also significantly promoting cell apoptosis. Additionally, these modulations significantly suppressed in vivo tumour growth.

**Conclusions:**

Circ_0078767 could suppress NSCLC progression by inhibiting miR‐330‐3p, which thereby increased RASSF1 levels.

## 1 INTRODUCTION

Lung cancer has been one of the main causes of death and one of the biggest health threats worldwide. According to the latest cancer statistics report in 2018, the incidence and mortality of lung cancer are 125.8 (2010‐2014) and 89.2 (2011‐2015) per 100 000 individuals, respectively, in the US population.^1^ The 5‐year relative survival rate of lung cancer is 18% in the United States (2007‐2013)^1^ and 19.7% in China (2012‐2015)^2^ and is among the lowest rates of all cancers. Non‐small‐cell lung cancer (NSCLC), including squamous cell lung carcinoma, lung adenocarcinoma and large cell lung carcinoma, accounts for over 75% of cases of lung cancer. Because of insufficient screening and a lack of early clinical symptoms, most NSCLC patients are diagnosed at late stages and have poor prognosis. As a result, the discovery of sensitive biomarkers and effective therapies are in critical demand.^3^

Ras‐related domain family member 1 transcript variant A (*RASSF1A*) is a well‐known tumour suppressor gene that is expressed in all normal human tissues and mediates the cell cycle and apoptosis.^4^ Daman reported that *RASSF1A* epigenetic inactivation in lung cancer indicated the role of *RASSF1A* as a lung tumour suppressor.^5^ *RASSF1A* was verified to be downregulated in NSCLC.^6^ *Rassf1a* knockout mice showed increased tumour multiplicity and size, which further established the tumour‐suppressive role of *Rassf1a*.^7^ In addition, it has been reported that RASSF1A exerts its tumour‐suppressing effects by inhibiting YAP activation,^8^ the Wnt signalling pathway^9^ and ACP‐Cdc20.^10^ However, as the molecular mechanism of *RASSF1A* participation in tumour progression has not been fully understood, more research on *RASSF1A* is needed.

MicroRNAs (miRNAs) are 21‐25 nucleotide small double‐stranded, non‐protein‐coding RNAs that can bind to the 3'‐UTR of target mRNAs and regulate gene transcription and translation by downregulating mRNA expression levels.^11^ miRNAs have been suggested to play significant roles in the progression of complex human diseases including cancers. Many studies have demonstrated that by targeting mRNAs, miRNAs exert their effects on cellular processes such as cell cycle regulation, differentiation, apoptosis, migration and invasion.^12^, ^13^ The functions of miRNAs have been observed in cellular processes and human diseases, such as macrophage phenotype and macrophage‐mediated tumour cell metastasis, endothelial cell junctions, cardiac dysfunction, osteoarthritis and vascular ageing.^14^, ^15^ Without a doubt, miRNAs also play an important role in NSCLC. Wang et al identified 35 upregulated miRNAs that were predicted to bind to at least one of 11 genes of the TGF‐β pathway as predictors of survival in advanced NSCLC, and 17 of the miRNAs were closely associated with patient survival in advanced NSCLC.^19^ Yu et al reported that miR‐193a inhibits NSCLC metastasis by downregulating the ERBB4/PIK3R3/mTOR/S6K2 signalling pathway.^20^ According to recent reports, miR‐21 was shown to promote growth, metastasis and chemotherapy or radiation resistance in NSCLC by targeting PTEN^21^ and modulate K‐Ras‐dependent lung tumorigenesis.^22^ Moreover, miR‐638 suppresses DNA damage repair, which affects cancer cell sensitivity to drugs.^23^ Increasing evidence has shown the importance of miRNAs as biomarkers and targets for novel therapies, and, as a result, a wider range of miRNAs should be thoroughly studied.

Long non‐coding RNAs (lncRNAs) have been frequently reported to be involved in NSCLC. For example, lncRNA NEAT1 has shown its ability to promote the progression of NSCLC,^24^ while lincRNA 0051 and lincRNA 319 promote NSCLC progression and lung adenocarcinoma carcinogenesis.^25^, ^26^ Circular RNAs (circRNAs), another member of the non‐coding RNA family, have been gradually explored in recent years. CircRNAs are a special type of RNA that form a covalently closed loop. These RNAs are common in eukaryotic cells and regulate gene transcription by functioning as efficient miRNA sponges and potent competing endogenous RNAs (ceRNAs).^27^, ^28^ Several reports have identified circRNAs to be potential biomarkers for cancers using microarray profiles; for example, circFARSA,^31^ circ_0014130,^32^ circ_0007385,^33^ and circFADS2,^34^ have been recognised as oncogenes in NSCLC. Additionally, circ_0007385 and circFADS2 have been confirmed to act as miRNA sponges.^33^, ^34^ The involvement of circRNAs has enhanced the chance of identifying biomarkers and potential novel targets, and thus more studies focused on circRNAs are urgently needed.

To address the potential roles of *RASSF1A*, miRNAs and the circRNA axis as well as their possible reciprocal relationship, we analysed existing microarray profiles, validated their dysregulation in NSCLC tissues and cell lines, and investigated their influence on lung cancer cell proliferation, apoptosis, invasion and cell cycle distribution. Furthermore, we verified their participation in lung cancer progression using a mouse model. To conclude, we found potential biomarkers for NSCLC and identified them as novel targets for new NSCLC therapies.

## 2 METHODS AND MATERIALS

### 2.1 Microarray and data analysis

CircRNA, miRNA and mRNA expression profiles for NSCLC tissues and adjacent normal tissues were generated using the Agilent microarray platform. The circRNA expression profile is accessible with accession number GSE101586. The miRNA and mRNA expression profiles are accessible with accession number GSE29250. Data processing and quantile normalisation were performed using R (Version 3.4.1) software. The calculated log2 (fold change) values (>1 or <−1) and *P* value (<0.05) were used to normalise the intensity and to set the threshold for the presence of human NSCLC RNA disorders.

### 2.2 Tissue samples

A total of 20 NSCLC tissues and 20 paired adjacent non‐tumour tissues (located 5 cm away from the tumour) were obtained from patients who underwent surgical resection at the Tumor Hospital of Yunnan Province. None of the patients had a history of tumours or received radiochemotherapy before surgery. This study was authorised by the Ethics Committee, and informed consent was signed by the enrolled patients before surgery. Immediately after resection, the tissue samples were frozen in liquid nitrogen and stored until use at −80°C. The clinical features of the patients are shown in Table 1.

**Table 1:** Correlation between expression of circ_0078767, miR‐330‐3p, RASSF1A and clinic pathological features in GC patients

Parameters	Group	Total	RASSF1A			miR‐330‐3p			circ_0078767		
			Low	High	*P*	Low	High	*P*	Low	High	*P*
Gender	Male	12	3	9	0.356	7	5	0.650	7	5	0.642
	Female	8	4	4		3	5		6	2	
Age	<60	6	3	3	0.613	2	4	0.629	5	1	0.354
	≥60	14	4	10		8	6		8	6	
Tumour size	<3 cm	5	4	1	0.037*	1	4	0.303	1	4	0.031*
	≥3 cm	15	3	12		9	6		12	3	
Differentiation	Well	8	4	4	0.356	3	5	0.650	6	2	0.642
	Moderate/Poor	12	3	9		7	5		7	5	
TNM stage	I + II	10	6	7	0.057	2	8	0.023*	9	1	0.057
	III + IV	10	1	3		8	2		4	6	
Lymph node metastasis	Absence	9	6	3	0.017*	8	1	0.005*	3	6	0.017*
	Presence	11	1	10		2	9		10	1	

Low and High expression group were divided according to the median ratio of relative RNA expression.

*P value was determined by chi‐square analysis. *P *＜ 0.05 was statistically significant.

### 2.3 Cell lines

Human NSCLC cell lines (A549, HCC827, NCI‐H23 and H125) and the normal human lung epithelial cell line BEAS‐2B were obtained from the BeNa culture collection. The A549 cell line was cultured in F12K (Gibco, Grand Island, NY, USA) with 10% foetal bovine serum (FBS; Gibco); HCC827, NCI‐H23 and H125 cell lines were maintained in RPMI1640 (Gibco) with 10% FBS (Gibco); and BEAS‐2B cell line was maintained in Dulbecco's modified Eagle's minimal essential medium (DMEM). All the cell lines were grown in a humidified incubator containing 5% CO_2_ at 37°C.

### 2.4 Quantitative real‐time reverse transcription PCR

The total RNA was extracted from cultured cells and fresh tissues using the TRIzol reagent (Invitrogen, Carlsbad, CA, USA) according to the manufacturer's instructions. Then, 500 ng of total RNA was reverse transcribed in a final volume of 10 µL with the Prime Script RT Master Mix (Takara, Japan). Quantitative real‐time reverse transcription PCR (qRT‐PCR) was executed using the SYBR Master Mix (ABI, Foster City, CA, USA) on an ABI 7500 system (ABI). RNA expression was quantified using the 2^−ΔΔCt^ method. The following primers were used for qRT‐PCR:

circ_0078767 (Forward), 5'‐GCCTAGCTGTCAAGGAGTGG‐3';

circ_0078767 (Reverse), 5'‐TCTAGAGATGCGCCAACACC‐3';

RASSF1A (Forward), 5'‐CCCCGCAGTGCTATTGCAT‐3';

RASSF1A (Reverse), 5'‐CACGAAGCGCACATTCTCTT‐3';

GAPDH (Forward), 5'‐GAAGGTGAAGGTCGGAGTC‐3';

GAPDH (Reverse), 5'‐GAAGATGGTGATGGGATTTC‐3';

miR‐330‐3p (Forward), 5'‐GCAGAGATTCCGTTGTCGT‐3';

miR‐330‐3p (Reverse), 5'‐GCGAGCACAGAATTAATACGAC‐3';

U6 (Forward), 5'‐AAAGCAAATCATCGGACGACC‐3';

U6 (Reverse), 5'‐GTACAACACATTGTTTCCTCGGA‐3'.

### 2.5 Plasmid construction, oligonucleotide synthesis and transfection

GenePharma (Shanghai, China) synthesised all the oligonucleotides. A specific siRNA for circ_0078767 was designed to target the covalent closed junction. The pcDNA3.1 plasmid containing circ_0078767 siRNA (sequence: 5'‐GTGAACGAGGCGGCTGTGGCG‐3') was constructed and used to knock down circ_0078767 expression. miR‐330‐3p mimics and an miR‐330‐3p inhibitor were used to enhance or downregulate miR‐330‐3p expression. The cells were transfected with 50 pmol/mL miR‐330‐3p mimic, negative control (NC) mimic, miR‐330‐3p inhibitor or NC inhibitor using Lipofectamine 2000 (Invitrogen) according to the manufacturer's protocol. The medium was substituted with culture medium at 6 hour after transfection. RASSF1A cDNA without its 3'‐UTR was inserted into the pcDNA3.1(+) vector (Invitrogen) to generate the recombinant RASSF1A vector and the empty vector (pcDNA3.1) was used as a control.

### 2.6 Cell proliferation assay

CCK‐8 analysis was performed 48 hours after transfection. The A549 and NCI‐H23 cell lines were seeded into 96 well sets at a density of 10^4^ cells/well. Cell viability was assessed at 0, 24, 48, 72 and 96 hours using the Cell Counting Kit (CCK)‐8 (Dojindo Molecular Technologies, Mashiki, Japan). The absorbance was measured at 450 nm using an Infinite M200 plate reader (Tecan, Männedorf, Switzerland).

### 2.7 Cell cycle and apoptosis analysis

For the cell cycle analysis, at 48 hour posttransfection, the cells (2 × 10^5^ cells) were digested by trypsin, washed twice with PBS, and fixed overnight at 4°C in 70% ethanol. Then, the cells were washed with PBS, centrifuged at 251.55 x *g* for 5 minutes and then treated with RNase A (0.1 mg/mL) and propidium iodide (PI, 0.05 mg/mL; Sigma, St Louis, MO, USA) for 20 minutes at room temperature. Cell cycle analysis was performed via FACS flow cytometry (BD Biosciences, Franklin Lakes, NJ, USA). To measure cell apoptosis, the cells were seeded in 6‐well plates (6 × 10^5^ cells/well) and cultured for 24 hour. Then, the cells were transfected and further incubated for 48 hour. The cells were washed twice with cold PBS and stained using the Annexin V/PI detection kit (BD Biosciences). Flow cytometry (BD Biosciences) was used to detect apoptotic cells according to the manufacturer's instructions. All experiments were carried out three times.

### 2.8 Transwell assay

Invasion assays were executed using a Transwell chamber with 8‐μm pores (Costar, Corning, New York, NY, USA). A Matrigel film (BD Biosciences) was used to coat the inserted top side for the invasion assay. The process chamber contained 200 μL serum‐free medium inoculated with 10^4^cells and the lower chamber contained 600 μL medium with 5% FBS. The chambers were maintained at 37°C and 5% CO_2_ for 24 hours. Afterwards, the non‐migrated or uninvaded cells on top of the membrane were removed with a cotton swab. The inserts were then fixed for 20 minutes in methanol and stained with 1% crystal violet for 30 minutes. The cells that migrated or invaded to the bottom of the membrane were studied and photographed under a microscope. All experiments were repeated three times.

### 2.9 Luciferase activity assay

A circRNA_0078767 segment was synthesised with either a mutant or a wild‐type (wt) seed region and cloned into the psiCHECK‐2 vector (ABI). The A549 and NCI‐H23 cell lines (1 × 10^5^ cells/well) were cotransfected with wild‐type or mutated (mut) circ_0078767 and miR‐330‐3p mimic or mimic control using Lipofectamine 2000 (Invitrogen). After induction for 48 hour, luciferase activity was assessed using the dual‐luciferase reporter kit (Promega, Madison, WI, USA). A wild‐type 3'‐UTR fragment of RASSF1A mRNA containing the putative miR‐330‐3p binding site was amplified by PCR and cloned downstream of the firefly luciferase gene in the pMIR‐REPORT vector (Thermo Scientific) to produce the pMIR‐RASSF1A‐3'UTR luciferase vector (RASSF1A‐wt). The QuikChange Mutagenesis kit (Stratagene, Palo Alto, CA, USA) was used to generate the mutant seed sequences and the mutated RASSF1A‐3'‐UTR fragment was cloned into pMIR‐REPORT vector to produce the pMIR‐RASSF1A‐3'UTR‐mut vector (RASSF1A‐mut). For the luciferase assay in A549 and NCI‐H23 cells, the cells were cotransfected in 48‐well plates with RASSF1A‐WT or RASSF1A‐MUT and miR‐330‐3p mimics or NC mimics using the Lipofectamine 2000 reagent (Invitrogen).

### 2.10 RNA immunoprecipitation

Biotin‐labelled circ_0078767 probe was synthesised by Sangon Biotech and the RNA immunoprecipitation (RIP) measurements were performed as described previously with slight modifications. A549 cells were fixed for 10 minutes with 1% formaldehyde, lysed and sonicated. After centrifugation, 50 μL of the supernatant was reserved as input, and the rest was incubated overnight at 30°C with a circ_0078767‐specific probe‐streptavidin dynabead (M‐280; Invitrogen) mixture. The next day, the M‐280 dynabeads‐probes‐circRNA mixture was washed with 200 μL lysis buffer and incubated with proteinase K before formaldehyde crosslinking was reversed. Finally, the RNA was extracted with TRIzol and RT‐qPCR detection was performed.

### 2.11 Western blotting

The total proteins were collected from cells using RIPA Lysis Buffer (Beyotime, Shanghai, China), and the protein concentration was measured with a BCA protein assay Kit (BioRad, Hercules, CA, USA). Equal amounts of protein (20 mg) were resolved by sodium dodecyl sulphate‐polyacrylamide gels and then transferred onto PVDF membranes (Millipore, Bedford, MA, USA). After blocking at room temperature with 5% skim milk for 1 hour, the membrane was incubated with the following primary antibodies: rabbit anti‐human RASSF1A antibody (ab97749, 1:2000; Abcam, Cambridge, MA, USA) and rabbit anti‐human GADPH antibody (ab22555, 1:1000; Abcam) at 4°C overnight. The membranes were then washed with TBST and incubated with HRP‐conjugated goat anti‐rabbit secondary antibody (ab97105, 1:5000; Abcam) at room temperature for 1 hour. The signal was detected using an enhanced chemiluminescence system (Thermo Scientific).

### 2.12 Immunohistochemistry

Immunohistochemistry (IHC) was performed as previously described. Formalin‐fixed and paraffin‐embedded tissue sections were incubated with Ki67 primary antibody (dilution 1:100; Santa Cruz). The slides were evaluated by two independent observers and scored on a scale from 0‐3 as follows: 0, absent positive tumour cells; 1, weak cell staining or <10% positive cells; 2, moderate cell staining or 10‐50% positive cells; and 3, intense cell staining or >50% positive cells.

### 2.13 Animal studies

All animal experiments were authorised by the Institutional Research Medical Ethics Committee at the Tumor Hospital of Yunnan Province. The 4‐6‐week‐old male nude mice (n = 15) were purchased from the Beijing Vital River Laboratory Animal Technology Co., Ltd. (Beijing, China) and randomly divided into five groups (NC group, circ_0078767 group, si‐circ_0078767 group, cotransfected si‐circ_0078767 and miR‐330‐3p inhibitor group and cotransfected si‐circ_0078767 and RASSF1A group) with three mice per group. Tumour xenografts were established by subcutaneously injecting 100 μL of corresponding cells (5 × 10^6^) and 30% Matrigel. The tumour sizes were measured every 5 days and tumour volumes were calculated using the following equation: 0.5 × length × width^2^. After 30 days, the mice were sacrificed, and the tumours were harvested, measured and weighted, as described previously.

### 2.14 Statistical analysis

The data are presented as the means ± SD from at least three independent experiments and were analysed using the SPSS 16.0 software (IBM, Armonk, NY, USA). Differences among the groups in the proliferation, cell cycle and apoptosis assays were determined using Student's *t* test. The paired samples *t* test was used to compare the differences between the tumour and non‐tumour tissues. The Pearson correlation coefficient was used to evaluate the correlation between any two molecules in the NSCLC tissues. A *P* value <0.05 was considered to be statistically significant.

## 3 RESULTS

### 3.1 RASSF1A was silenced in NSCLC tissues and cell lines

**Figure 1:**
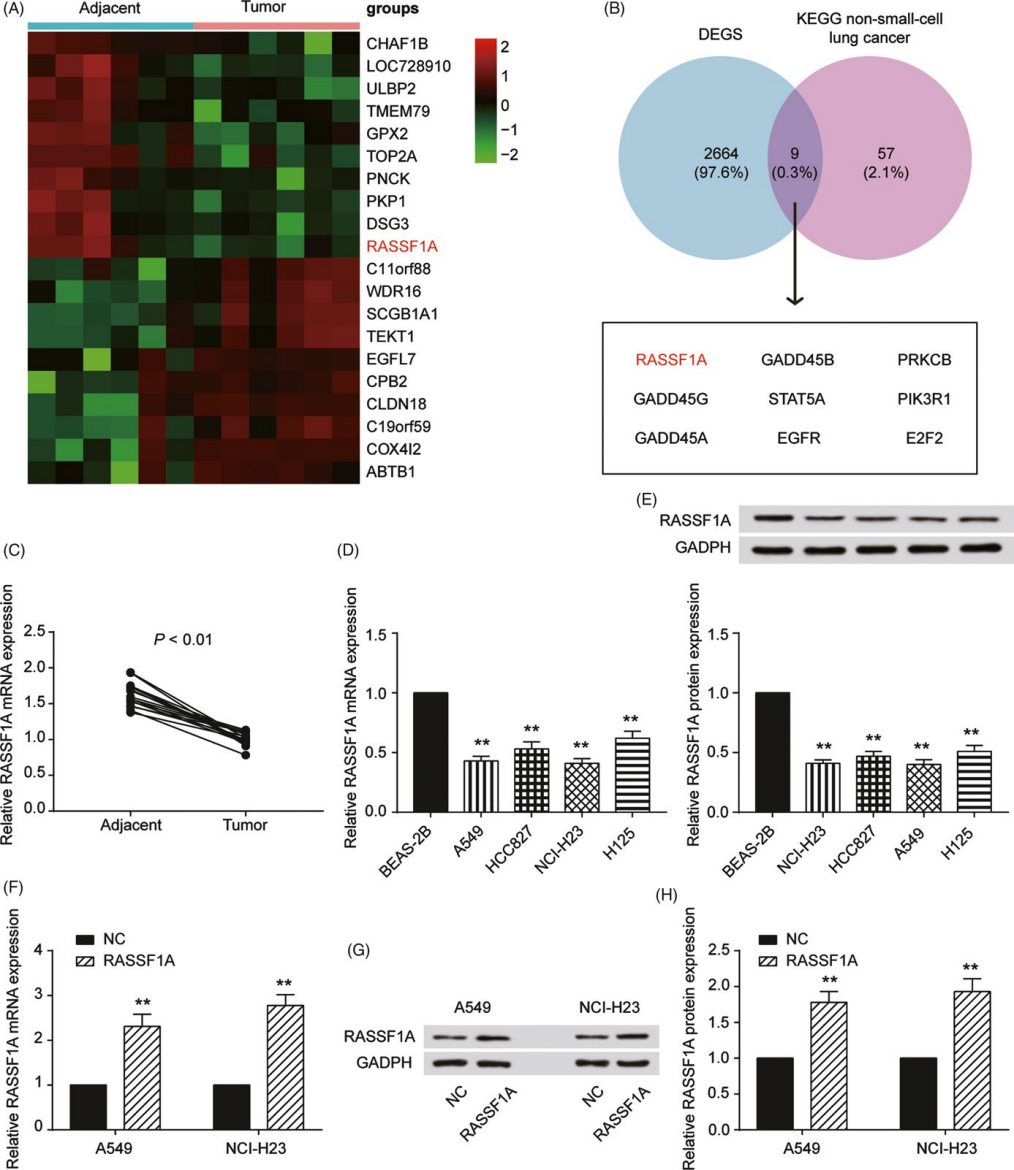
Characterisation of RASSF1A expression and construction of the abnormally expressed non‐small‐cell lung cancer (NSCLC) cell lines. A, Heat map: Among 20 genes, RASSF1A was significantly lowly expressed in cervical cancer tissues compared with that of adjacent tissues. B, Venn diagram of two sets: DEGS and KEGG non‐small‐cell lung cancer. RASSF1A was chosen from nine intersections. C, qRT‐PCR results showed that RASSF1A expression in tumours was significantly lower than that in adjacent tissues. D, RASSF1A expression in the NSCLC and normal cell lines by qRT‐PCR. E, Western blot results for RASSF1A expression in NSCLC and normal cell lines. F, RASSF1A was overexpressed in A549 and NCI‐H23 cell lines by RT‐qPCR. G, RASSF1A was overexpressed in A549 and NCI‐H23 cell lines by Western blot. H, The Western blot results in a bar graph. The data are from one representative experiment among three that were performed identically and are expressed as the means ± SD. **P < 0.01

Microarray analysis was applied to identify the differentially expressed mRNAs in NSCLC and adjacent normal tissues. There were 2673 differentially expressed mRNAs, and the top 10 upregulated and downregulated mRNAs are shown in Figure 1A. The intersection of the differentially expressed mRNAs and the genes involved in the KEGG NSCLC pathway included 9 mRNAs (RASSF1, GADD45B, PRKCB, GADD45G, STAT5A, PIK3R1, GADD45A, EGFR and E2F2). RASSF1 was among the top 10 downregulated mRNAs in tumour tissues (Figure 1B). We also examined the expression levels of RASSF1A in the tumour and adjacent tissues, and the results showed that RASSF1A mRNA (*P *< 0.01, Figure 1C) and protein (Supplementary Figure A) levels were significantly lower in tumour tissues than those in adjacent tissues. The same results were revealed in cell experiments. RASSF1A mRNA and protein expression were significantly decreased in all four NSCLC cell lines compared with that of the human lung epithelial cell line BEAS‐2B (*P *< 0.01, Figure 1D,E). We concluded that RASSF1A might be involved in inhibiting NSCLC tumorigenesis and performed in vitro experiments in A549 and NCI‐H23 cell lines.

### 3.2 RASSF1A functions in NSCLC cell lines

**Figure 2:**
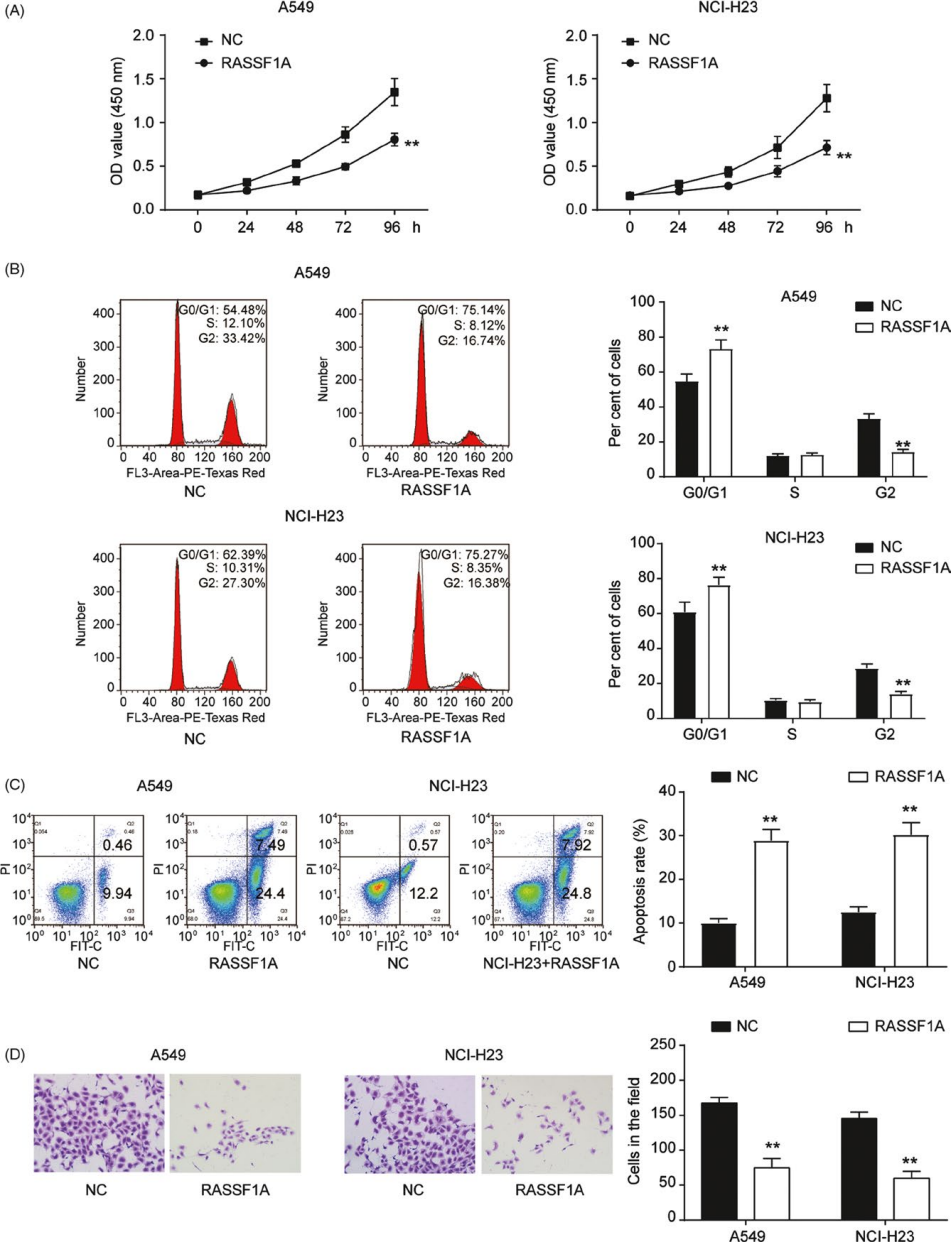
RASSF1A function in non‐small‐cell lung cancer (NSCLC) cell lines. A, CCK‐8 results showed that RASSF1A overexpression inhibited cell proliferation. B, Transfection of NSCLC cell lines with RASSF1A resulted in G0/G1 phase arrest by FACS flow cytometry. C, RASSF1A overexpression increased apoptotic cells by flow cytometry. D, Transwell assay showed that RASSF1A inhibited cell invasion. The data are from one representative experiment among three that were performed identically and are expressed as the means ± SD. **P < 0.01

RASSF1A functions were analysed by examining proliferation, cell‐cycle progression, apoptosis and invasion using the A549 and NCI‐H23 cell lines. The upregulation of RASSF1A in A549 and NCI‐H23 cells significantly increased RASSF1A mRNA and protein expression (*P *< 0.01, Figure 1F‐H). To test the effects of RASSF1A on cell proliferation, we employed the CCK‐8 assay. RASSF1A was overexpressed in the different cell lines, and the number of viable cells was assessed for up to 96 hour. The overexpression of RASSF1A inhibited the growth of A549 and NCI‐H23 cells (*P *< 0.01, Figure 2A). For cell cycle progression, RASSF1A upregulation in the two cell lines resulted in a significant increase in the number of cells in the G0/G1 phase of the cell cycle and a concomitant reduction in the number of cells in S phase, suggesting that RASSF1A may play a role in G0/G1 exit (*P *< 0.01, Figure 2B). Furthermore, the apoptosis analysis indicated that RASSF1A played a role in enhancing apoptosis (*P *< 0.01, Figure 2C). A transwell assay was also executed to examine cell invasion. The results showed that the number of cells that invaded through the chamber in the RASSF1A‐overexpressing group was significantly lower than that in the NC group, indicating that RASSF1A inhibited NSCLC cell invasion (*P *< 0.01, Figure 2D). The functional experiments showed that RASSF1A expression inhibited NSCLC tumorigenesis.

### 3.3 miR‐330‐3p suppressed NSCLC proliferation by inhibiting RASSF1A expression

**Figure 3:**
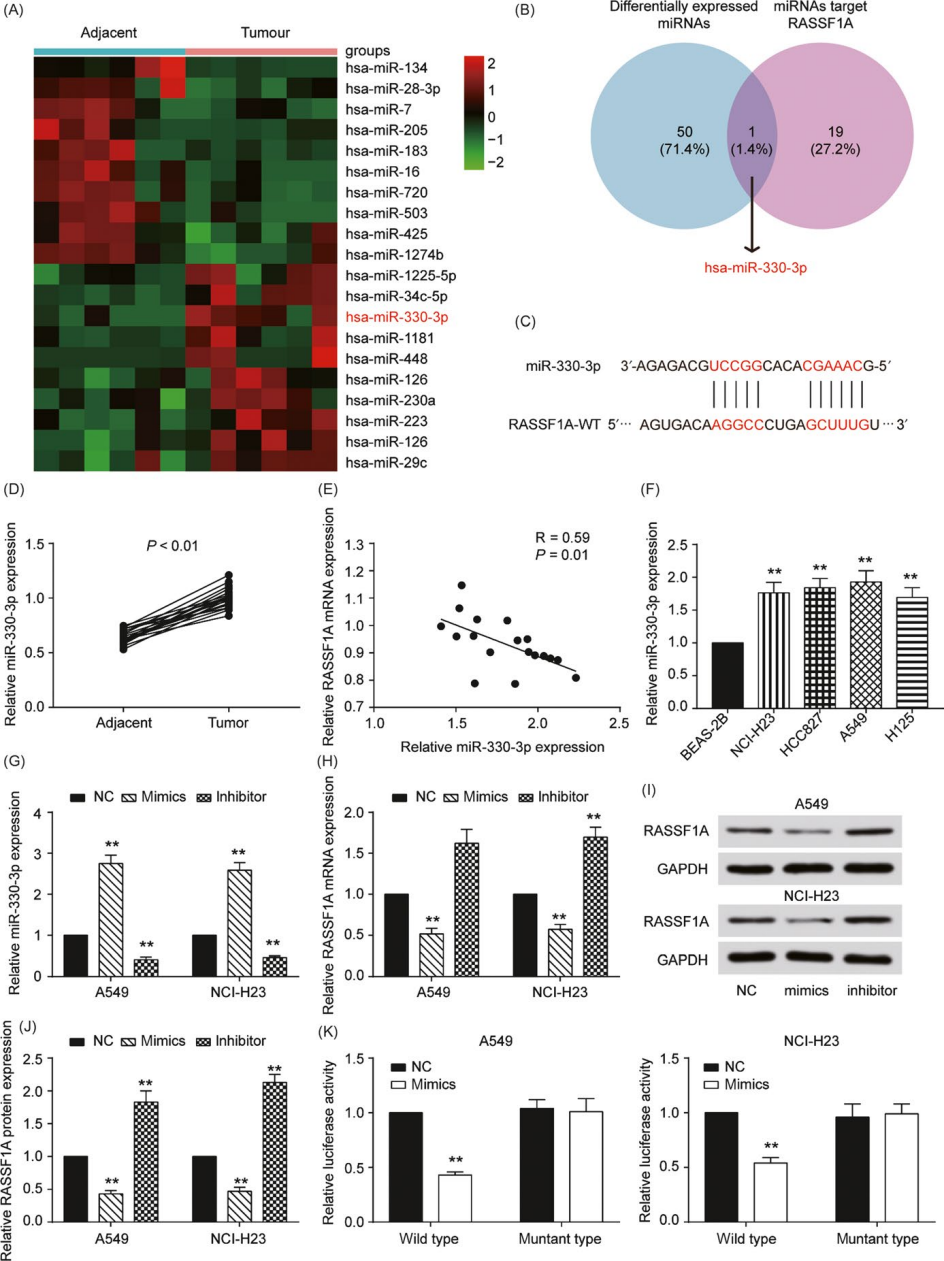
RASSF1A was targeted by miR‐330‐3p. A, Heat map: Among 20 microRNAs, miR‐330‐3p was significantly highly expressed in cervical cancer tissues compared with that in adjacent tissues. B, Venn diagram of two sets: Differentially expressed miRNAs and miRNAs targeting RASSF1A. miR‐330‐3p was chosen from nine intersections. C, The estimated RASSF1A binding sites for miR‐330‐3p. D, qRT‐PCR results showed that miR‐330‐3p expression in tumours was significantly higher than that in adjacent tissues. E, RASSF1A expression was negatively correlated with miR‐330‐3p in non‐small‐cell lung cancer (NSCLC) tissues. *R* = 0.59, *P* = 0.01. F, qRT‐PCR results showed that miR‐330‐3p expression in NSCLC cell lines was significantly higher than that in normal human lung epithelial cell lines. G, qRT‐PCR results showed that miR‐330‐3p was upregulated by miR‐330‐3p mimics and inhibited by miR‐330‐3p inhibitors. H, RASSF1A was upregulated by miR‐330‐3p mimics and inhibited by miR‐330‐3p inhibitors by qRT‐PCR. I, RASSF1A was upregulated by miR‐330‐3p mimics and inhibited by miR‐330‐3p inhibitors by Western blot. J, The Western blot results in a bar graph. K, miR‐330‐3p decreased the luciferase activity of the RASSF1A wild‐type 3'‐UTR. The data are from one representative experiment among three that were performed identically and are expressed as the means ± SD. **P < 0.01

**Figure 4:**
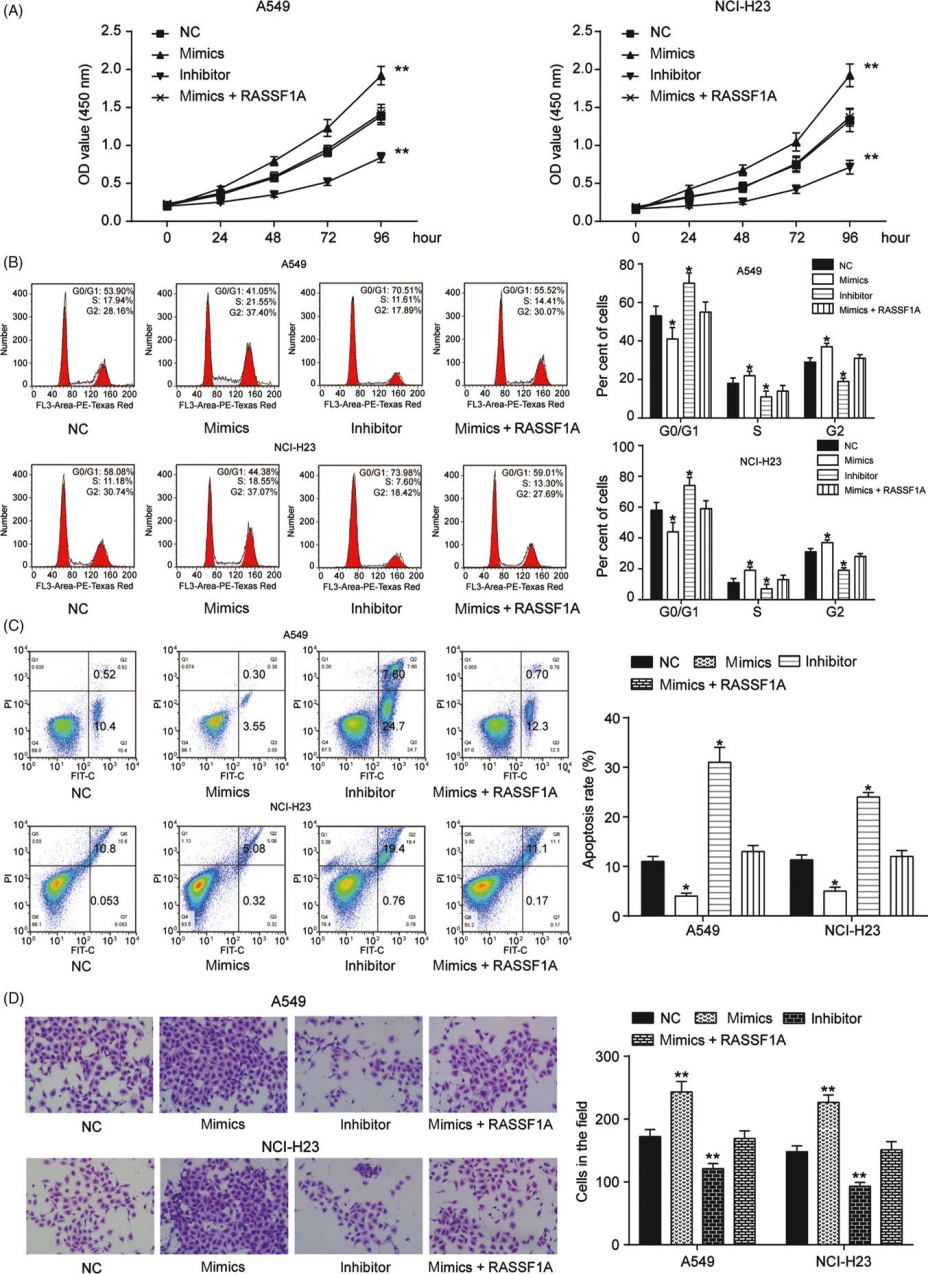
MiR‐330‐3p mimics abrogated the function of RASSF1A knockdown in non‐small‐cell lung cancer (NSCLC) cell lines. A, Cell proliferation assay was evaluated by CCK‐8 assay. In A549 and NCI‐H23 cell lines, miR‐330‐3p inhibitor suppressed cell proliferation, whereas miR‐330‐3p mimics promoted it. Co‐transfection of mimics and RASSF1A inhibited this promotion. B,Transfection of NSCLC cell lines with miR‐330‐3p resulted in G0/G1 phase arrest by FACS flow cytometry. C, The apoptotic cells in the mimics group, inhibitor group and mimics + RASSF1A group were measured by flow cytometry. D, MiR‐330‐3p mimics increased cell invasion, which was reversed by RASSF1A. The data are from one representative experiment among three that were performed identically and are expressed as the means ± SD. *P < 0.05 and **P < 0.01

We next determined the molecular mechanism that regulated RASSF1A and influenced NSCLC. The microarray analysis showed that there were 51 differentially expressed miRNAs in NSCLC, and the top 10 upregulated and downregulated miRNAs are shown in Figure 3A. We found 20 miRNAs that targeted RASSF1A, though only miR‐330‐3p was involved in both groups (Figure 3B,C). qRT‐PCR indicated that miR‐330‐3p was upregulated in both NSCLC tissues and cell lines (compared with that of adjacent tissue, *P *< 0.01, Figure 3D,F). We also found that miR‐330‐3p expression was negatively correlated in the tumour tissues (Figure 3E). Furthermore, we transfected miRNA mimics and inhibitors to regulate the expression of miR‐330‐3p (*P *< 0.01, Figure 3G). qRT‐PCR and Western blotting revealed that RASSF1A expression was significantly decreased by the mimics and increased by the inhibitors (Figure 3H‐J). We then performed a dual‐luciferase reporter assay and verified that miR‐330‐3p targeted RASSF1A (*P *< 0.01, Figure 3K). Eventually, we measured cell proliferation as regulated by miR‐330‐3p and RASSF1A by CCK‐8 assay. The mimics upregulated cell growth in both A549 and NCI‐H23 cells, whereas the inhibitors suppressed cell growth. Moreover, co‐transfection of miR‐330‐3p mimics and RASSF1A reversed the proliferation induced by the mimics (*P *< 0.01, Figure 4A). For cell cycle progression, the transfection of miR‐330‐39 mimics into the cell lines resulted in a significant decrease in the number of cells in the G0/G1 phase of the cell cycle and a concomitant increase in the number of cells in S phase, suggesting that miR‐330‐39 may play a role in G0/G1 exit (*P *< 0.01, Figure 4B). Furthermore, the apoptosis analysis indicated that miR‐330‐3p played a role in restraining apoptosis (*P *< 0.01, Figure 4C). Cell invasion assays showed the same results (*P *< 0.01, Figure 4D), suggesting that the functions of miR‐330‐3p and RASSF1A in NSCLC are antagonistic.

### 3.4 circ_0078767 inhibits miR‐330‐3p and reverses the suppression of RASSF1A

**Figure 5:**
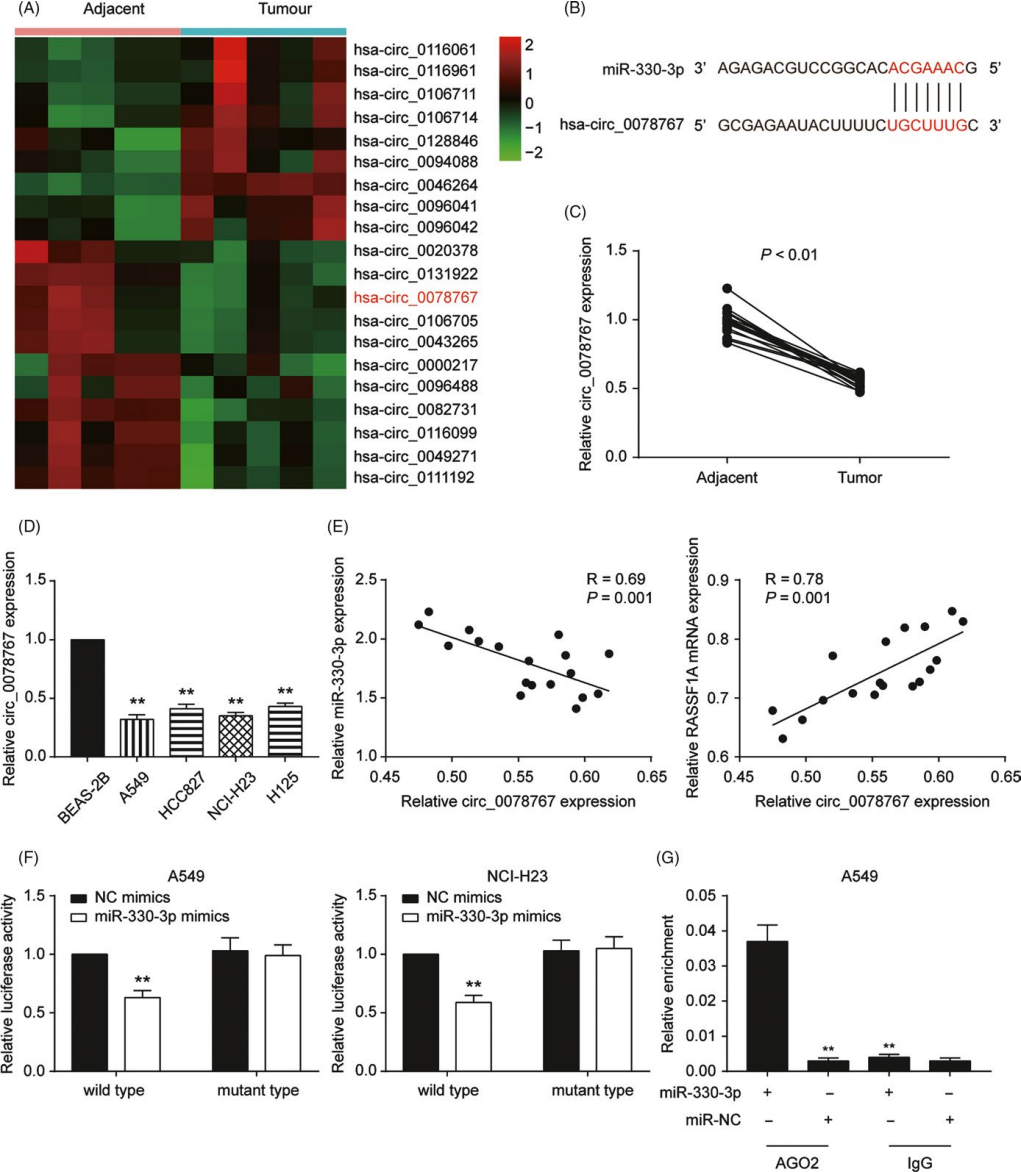
Circ_0078767 directly binds to miR‐330‐3p and suppresses miR‐330‐3p activity. A, Heat map: Among 20 circRNA, circ_0078767 was significantly lowly expressed in cervical cancer tissues compared with that in adjacent tissues. B, The estimated circ_0078767 binding sites for miR‐330‐3p. C, qRT‐PCR results showed that circ_0078767 expression in tumours was significantly higher than in adjacent tissues. D, qRT‐PCR results showed that circ_0078767 expression in non‐small‐cell lung cancer (NSCLC) cell lines was significantly lower than that in normal human lung epithelial cell lines. E, circ_0078767 expression was negatively correlated with miR‐330‐3p (*R* = 0.69, *P* = 0.001) and positively correlated with RASSF1A (*R* = 0.78, *P* = 0.001) in NSCLC tissues. F, miR‐330‐3p decreased the luciferase activity of the wild‐type circ_0078767 3'‐UTR. G, Anti‐AGO2 RIP was performed in the A549 cell line transfected with miR‐330‐3p mimics or negative control (NC), followed by qRT‐PCR to detect circ_0078767. The data are from one representative experiment among three that were performed identically and are expressed as the means ± SD. **P < 0.01

**Figure 6:**
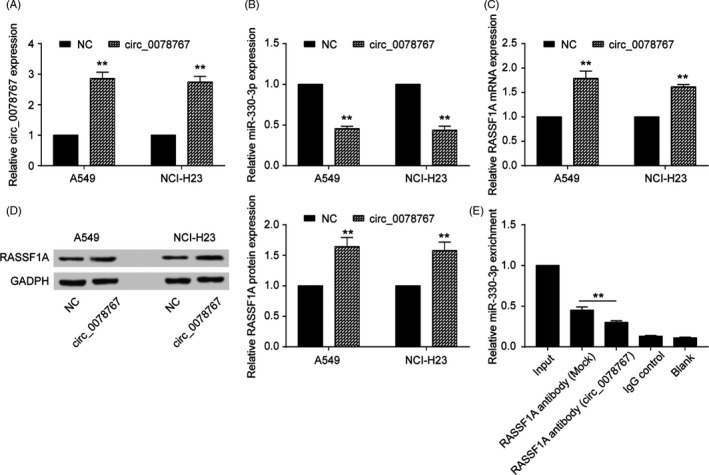
Circ_0078767 increased RASSF1A expression by inhibiting miR‐330‐3p. A, Circ_0078767 was increased by the transfection of the circ_0078767 vector in both non‐small‐cell lung cancer (NSCLC) cell lines. B, NSCLC cell lines were transfected with circ_0078767, and the subsequent downregulation of miR‐330‐3p expression was detected via qRT‐PCR. C, qRT‐PCR results showed that RASSF1A was upregulated by circ_0078767. D, Western blot results showed that RASSF1A was upregulated by circ_0078767. E, RIP experiments were performed using the anti‐RASSF1A antibody to immunoprecipitate RNA and a primer to detect miR‐330‐3p. Significantly decreased enrichment of miR‐330‐3p was identified in cells transfected with the circ_0078767‐overexpressing vector. The data are from one representative experiment among three that were performed identically and are expressed as the means ± SD. **P < 0.01

We next studied the potential targeting of miR‐330‐3p by circRNAs using a bioinformatic assay, and circ_0078767 was chosen as the targeting circRNA for the subsequent experiments (Figure 5A‐B). qRT‐PCR results showed that circ_0078767 was significantly downregulated in NSCLC tissues and cell lines (Figure 5C,D). We then found that circ_0078767 expression was positively correlated with miR‐330‐3p, which is negatively correlated with RASSF1A (*P *< 0.01, Figure 5E). We further explored the interaction between circ_0078767 and miR‐330‐3p. We used a dual‐luciferase reporter assay in A549 and NCI‐H23 cells to investigate whether circ_0078767 functionally targeted miR‐330‐3p. The results verified this relationship (*P *< 0.01, Figure 5F). It is known that miRNAs repress translation and degrade mRNAs in an AGO2‐dependent manner by binding to their targets. We conducted anti‐AGO2 immunoprecipitation (RIP) with anti‐AGO2 or control IgG antibodies in cervical cells and cervical cells transiently overexpressing miR‐506 to pull down circ‐0078767, followed by RT‐qPCR analysis for circ_0078767 levels. The results showed that circ_0078767 pulled down with anti‐AGO2 antibodies was significantly enriched in cells transfected with miR‐330‐3p mimics compared to that of the controls (*P *< 0.01, Figure 5G). For the functional assay, we overexpressed circ_0078767 and verified its expression by qRT‐PCR (*P *< 0.01, Figure 6A). In addition, the overexpression of circ‐0078767 significantly inhibited miR‐330‐3p in NSCLC cell lines (*P *< 0.01, Figure 6B), whereas RASSF1A was significantly promoted (*P *< 0.01, Figure 6C,D). Furthermore, an RIP assay showed that the enrichment of RASSF1A and miR‐330‐3p was significantly decreased in A549 cells transfected with the circ_0078767 vector (Figure 6E), suggesting that circ_0078767 sponges miR‐330‐3p, which subsequently allows for RASSF1A translation.

### 3.5 circ_0078767 suppresses cell proliferation and invasion via the miR‐330‐3p/RASSF1A pathway

**Figure 7:**
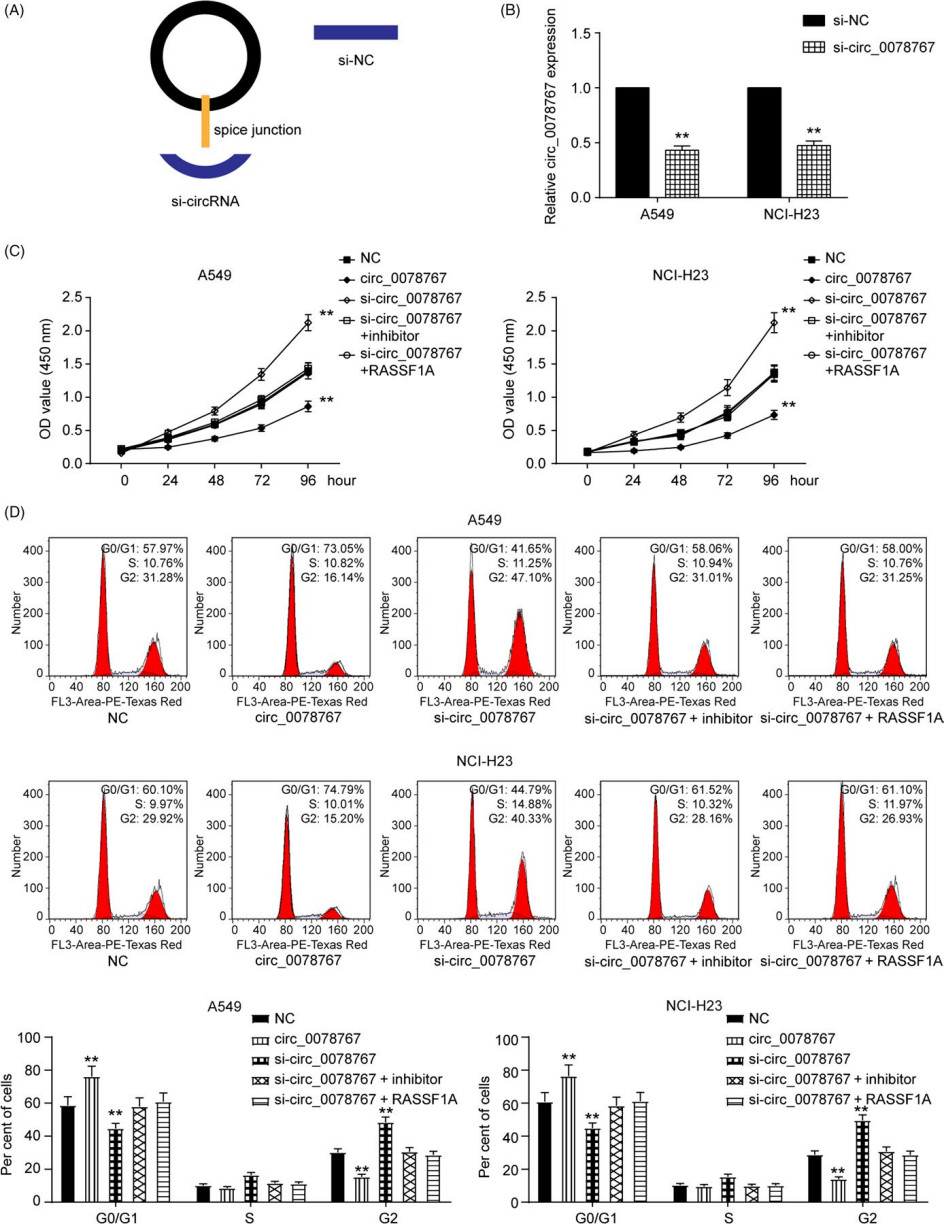
Circ_0078767 regulates cell proliferation and cell cycle via the miR‐330‐3p/RASSF1A pathway. A, The structures of si‐circRNA and si‐NC vector are shown. B, Circ_0078767 was silenced by the transfection of specific silencing vectors in both cervical cell lines. C, CCK‐8 assay showed that inhibiting circ_0078767 expression promoted the cell proliferation rate of both cell lines; however, co‐transfection of an miR‐330‐3p inhibitor or RASSF1A significantly reversed this effect. D, FACS flow cytometry assay suggested that the alleviated G0/G1 phase arrest of both cell lines induced by si‐circ_0078767 was significantly abrogated by co‐transfection with an miR‐330‐3p inhibitor or RASSF1A. The data are from one representative experiment among three that were performed identically and are expressed as the means ± SD. **P < 0.01

**Figure 8:**
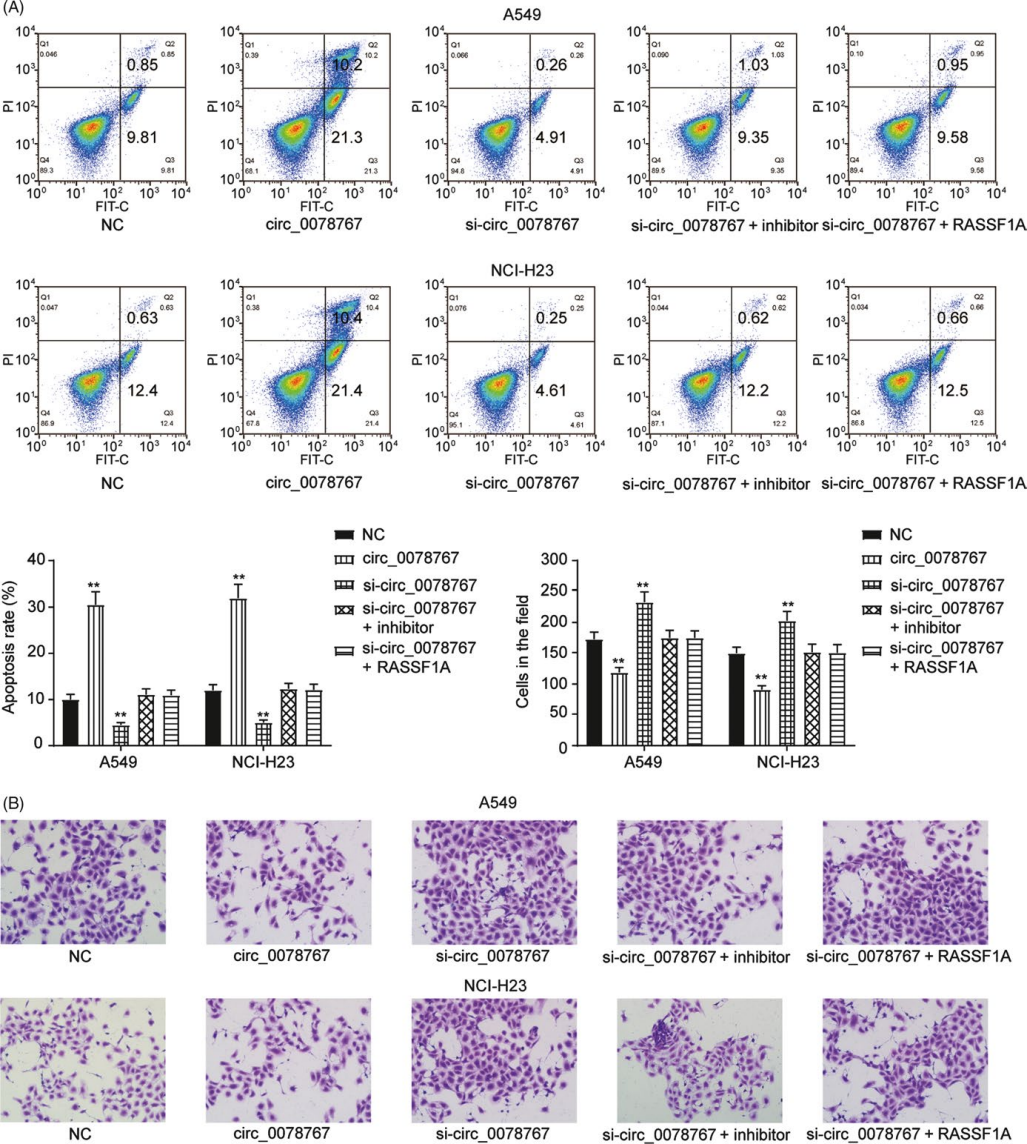
Circ_0078767 regulated apoptosis and cell invasion via the miR‐330‐3p/RASSF1A pathway. A, Flow cytometry assay showed that inhibiting circ_0078767 expression downregulated the cell apoptosis rate in both cell lines; however, co‐transfection of an miR‐330‐3p inhibitor or RASSF1A significantly reversed this effect. B, Cell invasion assay suggested that the increased cell invasion of non‐small‐cell lung cancer (NSCLC) cell lines induced by si‐circ_0078767 was abrogated by co‐transfection with miR‐330‐3p inhibitor or RASSF1A. The data are from one representative experiment among three that were performed identically and are expressed as the means ± SD. **P < 0.01

Based on the above observations, we further detected the function of circ_0078767 in NSCLC. The full‐length cDNA of circ_0078767 from NSCLC was amplified and cloned into a specific vector (Figure 7A). qRT‐PCR showed that si‐circ_0078767 significantly downregulated circ_0078767 levels in both cell lines (*P *< 0.01, Figure 7B). Furthermore, the inhibition of circ_0078767 expression promoted cell proliferation; however, co‐transfection of an miR‐330‐3p inhibitor or a RASSF1A vector significantly reversed this effect (*P *< 0.01, Figure 7C). Similarly, the decreased number of cells in the G0/G1 phase (*P *< 0.01, Figure 7D) inhibited the apoptosis rate (*P *< 0.01, Figure 8A) and enhanced the cell invasive capacity associated with NSCLC (*P *< 0.01, Figure 8B), which were all induced by si‐circ_0078767 and were abrogated due to co‐transfection with miR‐330‐3p inhibitor or RASSF1A vector.

### 3.6 Inhibition of the circ_0078767/miR‐330‐3p/RASSF1A axis enhances tumour progression in vivo

**Figure 9:**
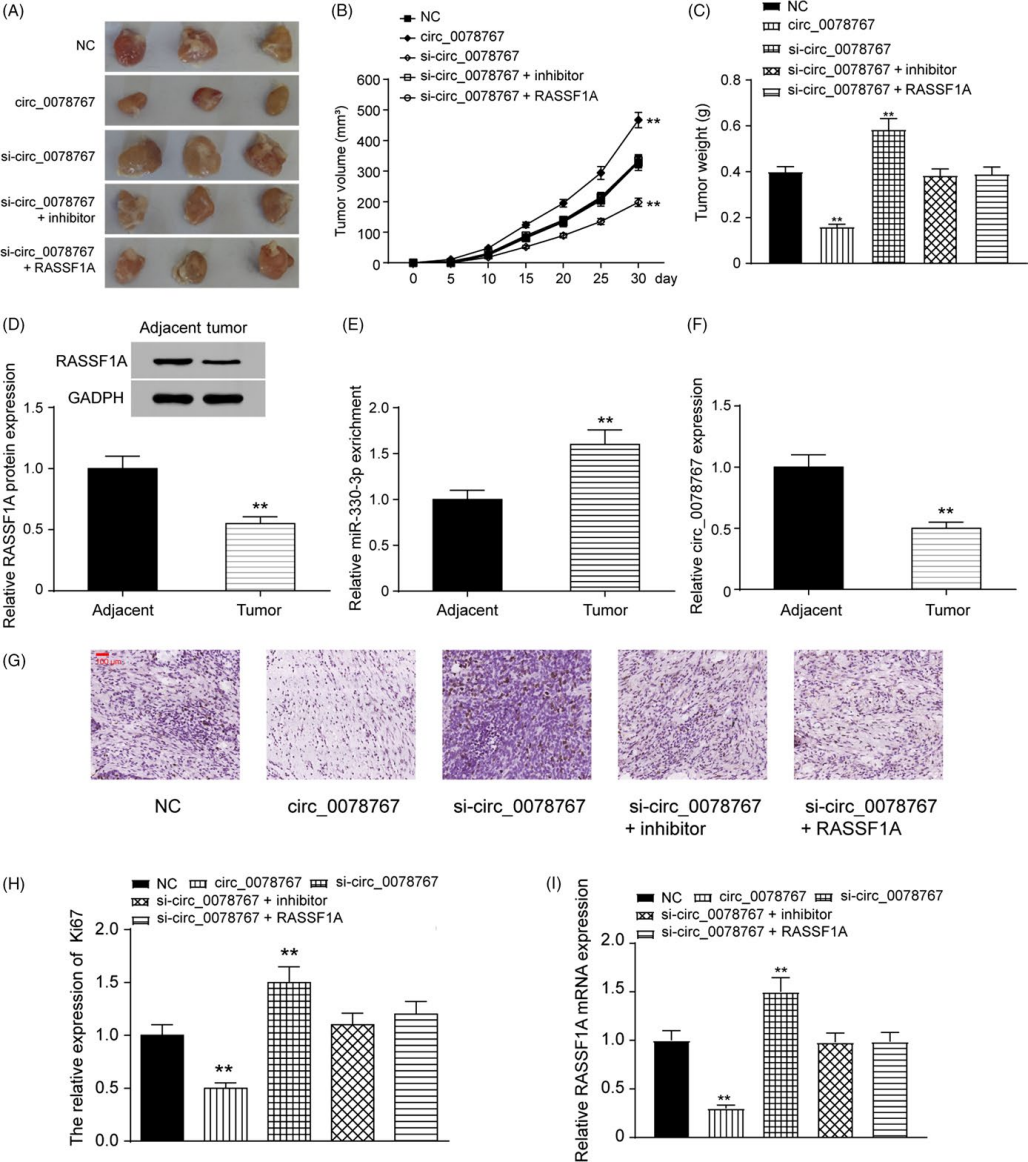
The circ_0078767/miR‐330‐3p/RASSF1A axis suppressed tumour formation in vivo. A, Representative tumours induced by circ_0078767, si‐circ_0078767 or cotransfected with miR‐330‐3p inhibitor or RASSF1A. B, Inhibiting circ_0078767 expression upregulated tumour volume; however, co‐transfection of an miR‐330‐3p inhibitor or RASSF1A significantly reversed this effect. C, The increased tumour weights induced by si‐circ_0078767 were abrogated by co‐transfection with miR‐330‐3p inhibitor or RASSF1A. D, RASSF1A in tumours from the animal model was measured by Western blot. E, qRT‐PCR results showed that miR‐330‐3p expression in tumours was significantly higher than that in adjacent tissues. F, qRT‐PCR results showed that circ_0078767 expression in tumours was significantly higher than that in adjacent tissues. G‐H, Ki67 was selected as a tumour marker and was measured by immunohistochemistry (I) RASSF1A mRNA was extracted from groups of tumours and analysed by qRT‐PCR. The data are from one representative experiment among three that were performed identically and are expressed as the means ± SD. **P < 0.01

To explore the effects of the circ_0078767/miR‐330‐3p/RASSF1A axis on NSCLC in vivo, we established the following five A549 cell groups: NC group, circ_0078767 group, si‐circ_0078767 group, cotransfected si‐circ_0078767 and miR‐330‐3p inhibitor group and cotransfected si‐circ_0078767 and RASSF1A group. All the A549 groups were inoculated into mice. As shown by the representative tumours, tumours overexpressing circ_0078767 in A549 cells grew quicker and had larger volumes than tumours in the vector group. Si‐circ_0078767‐induced promotion of tumorigenesis was reversed by co‐transfection with an miR‐330‐3p inhibitor or RASSF1A (Figure 9A). At the end of the experiments, the xenograft tumours were dissected, measured and weighed. The average tumour volume and weight of the circ_0078767 group was significantly higher than that of the vector group but lower than that of the si‐circ_0078767 group. The data for the NC group cotransfected si‐circ_0078767 and miR‐330‐3p inhibitor group and cotransfected si‐circ_0078767 and RASSF1A group showed no significant differences (*P *< 0.01, Figure 9B,C). As shown in Figure 9D, RASSF1A protein levels were lower in mouse tumour tissues than those in adjacent tissues. qRT‐PCR results showed that miR‐330‐3p expression in the tumours was significantly higher than that in adjacent tissues, while circ_0078767 expression was lower in the tumours than that in adjacent tissues (*P *< 0.01, Figure 9E,F). Ki67 was selected as a tumour marker and was measured by immunohistochemistry. The circ_0078767 group had the lowest Ki67 expression, while the si‐circ_0078767 group showed the highest expression (*P *< 0.01, Figure 9G,H). RASSF1A expression in the tumours was also analysed by qRT‐PCR (*P *< 0.01, Figure 9I). Taken together, these results indicate that the inhibition of the circ_0078767/miR‐330‐3p/RASSF1A axis promoted tumorigenesis in vivo.

## 4 DISCUSSION

In our study, we demonstrated the tumour‐suppressive role of RASSF1A and circ_0078767 and the oncogenic role of miR‐330‐3p in vitro and in vivo. We analysed the expression levels of RASSF1A and circ_0078767 using microarrays and measured them in lung cancer patients and cell lines; their effects on tumour progression were evaluated by examining cell proliferation, apoptosis, invasion and cell cycle distribution. A mouse xenograft model was also developed to assess the effects of RASSF1A, miR‐330‐3p and circ_0078767 on tumour growth.

In the present study, RASSF1A expression was decreased in tumour tissues compared to that of adjacent normal tissues. Upregulation of RASSF1A led to decreased cell proliferation, limited invasion, increased apoptosis and cell cycle arrest in the G0/G1 phase in lung cancer cell lines, which indicated impaired tumour malignancy. Dallol et al discovered that RASSF1A overexpression inhibits cell migration and changes cell morphology in the A549 lung cancer cell line.^35^ Duboiset et al reported that RASSF1A knockdown causes invasiveness, while restoring RASSF1A expression decreased A549 cell invasion in vitro.^8^ The results of our study were consistent with those of previous reports and verified the tumour‐suppressive effect of RASSF1A in lung cancer pathogenesis.

The anti‐inflammatory power of miRNAs is distinctly strong in NSCLC. As shown in previous studies, miR‐134 and miR‐187‐3p suppressed NSCLC progression^36^, ^37^ and miR‐9600 could sensitise NSCLC to paclitaxel sensitivity.^38^ In the study by Iaboni et al, aptamer‐miR‐212 conjugates sensitised NSCLC cells to TNF‐related apoptosis‐inducing Ligand.^39^ MiRNAs perform different roles in changing environments; for example, it has been reported that miR‐346 promotes cell growth and metastasis and inhibits apoptosis in NSCLC.^40^ Thus, the functions of miRNAs must be investigated in specific contexts to draw specific conclusions.

MiR‐330‐3p was confirmed by our study to target RASSF1A and displayed opposite expression levels to that of RASSF1A in lung cancer patients and cell lines. A miR‐330‐3p inhibitor successfully knocked down miR‐330‐3p expression and subsequently restored RASSF1A expression levels in lung cancer cell lines. The downregulation of miR‐330‐3p resulted in decreased cell proliferation and increased apoptosis and cell cycle arrest in the G0/G1 phase in lung cancer cell lines, which were relevant to attenuating lung cancer cell invasiveness. Additionally, the downregulation of miR‐330‐3p rescued lung cancer cell invasiveness and growth caused by RASSF1A knockdown. miR‐330‐3p has been investigated and reported as a potential prognostic biomarker in several diseases. Meng et al reported that miR‐330‐3p targeted the PDCD4 oncogene and played a role in human oesophageal cancer.^41^ Mesci et al reported that miR‐330‐3p promoted human breast cancer metastasis by targeting CCBE1^42^ and Wei et al reported that miR‐330‐3p promotes cell invasion and metastasis in NSCLC through GRIA3.^43^ In the present study, we validated that miR‐330‐3p functions as an oncogene, and investigated the probability that miR‐330‐3p promotes NSCLC by targeting RASSF1A.

Through our research, circ_0078767 was found to be lowly expressed in tumour tissues compared to that in adjacent normal tissues as well as a verified target of miR‐330‐3p. circ_0078767 overexpression caused a significant decrease in cell proliferation, limited invasion, increased apoptosis and cell cycle arrest in G0/G1 phase in lung cancer cell lines, indicating weakened lung cancer cell malignancy. In addition, circ_0078767 could also prevent the increase in lung cancer cell aggressiveness that resulted from miR‐330‐3p overexpression and RASSF1A knockdown, demonstrating its inhibition of miR‐330‐3p and, conversely, promotion of RASSF1A. In the mouse xenograft model, circ_0078767 showed a critical effect by shrinking tumour volume and inhibiting tumour growth due to miR‐330‐3p overexpression and RASSF1A knockdown. As a well‐known ceRNA, lncRNAs take part in cancer progression by targeting miRNAs. For example, lncRNA PDIA3P interacts with miR‐185‐5p to facilitate oral squamous cell carcinoma progression.^44^ CircRNAs were also predicted to act as ceRNA molecules; for example, circ_0007385 was recognised as an oncogene in NSCLC and proven to be able to target miR‐181^33^ by acting as a sponge for miR‐498; circFADS2 has also been proven to promote lung cancer cell proliferation and invasion.^34^ Previous studies assured the possibility that circ_0078767 could function as a miR sponge for miR‐330‐3p and therefore promote the tumour‐suppressive effects of RASSF1A, as well as its involvement in the circ_0078767/miR‐330‐3p/RASSF1A axis in NSCLC.

Despite our findings, more studies are needed to support the role of the circ_0078767/miR‐330‐3p/RASSF1A axis in NSCLC. RASSF1A should be further investigated along with its isoform RASSF1C, whose role in cancer pathogenesis is still under debate. More ceRNA molecules interacting with circ_0078767, miR‐330‐3p and RASSF1A should also be taken into consideration. Furthermore, the effects of miR‐330‐3p on RASSF1A at the transcriptional level are also worth exploring. In accordance with a recent study, magnetic nanocarriers have been applied to detect the accurate target of miRNAs in tumour cells, which would provide great convenience for future studies.^45^ To gain deeper insights into NSCLC progression, other dysregulated genomes and transcriptomes as well as related signalling pathways should be studied.

In summary, we reported the tumour‐suppressive role of RASSF1A and circ_0078767 as well as the oncogenic role of miR‐330‐3p in this study. We found that RASSF1A, miR‐330‐3p and circ_0078767 are potential biomarkers for NSCLC, and we identified them as novel targets for the treatment of NSCLC.

## CONFLICT OF INTEREST

Authors declare no conflict of interests for this article.

Informed Consent: Written informed consents were obtained from all individual participants included in the study.

## AUTHOR CONTRIBUTION

Ting Chen conceived and designed of the work and acquisition, analysed the data and drafted the article; Zuozhang Yang and Chao Liu analysed the data and drafted the article; Li Wang, Jun Yang and Long Chen performed the experiments and revised the article critically for important intellectual content; Wenhui Li analysed the data and revised the article critically for important intellectual content. All authors read and approved the version to be published, and participated sufficiently in the work to take public responsibility for appropriate portions of the content.

## ETHICAL APPROVAL

All procedures performed in studies involving human participants were in accordance with the ethical standards of Tumor Hospital of Yunnan Province, The Third Affiliated Hospital of Kunming Medical College committee.

## DATA AVAILABLITY

The data sets used and analysed during the current study are available from the corresponding author on reasonable request.
