# Supplementary figures and images for: Circ_0078767 suppresses non‐small‐cell lung cancer by protecting RASSF1A expression via sponging miR‐330‐3p
Source: Cell Prolif. 2018 Dec 3;52(2):e12548. doi: 10.1111/cpr.12548 (PMC6496360; doi:10.1111/cpr.12548)

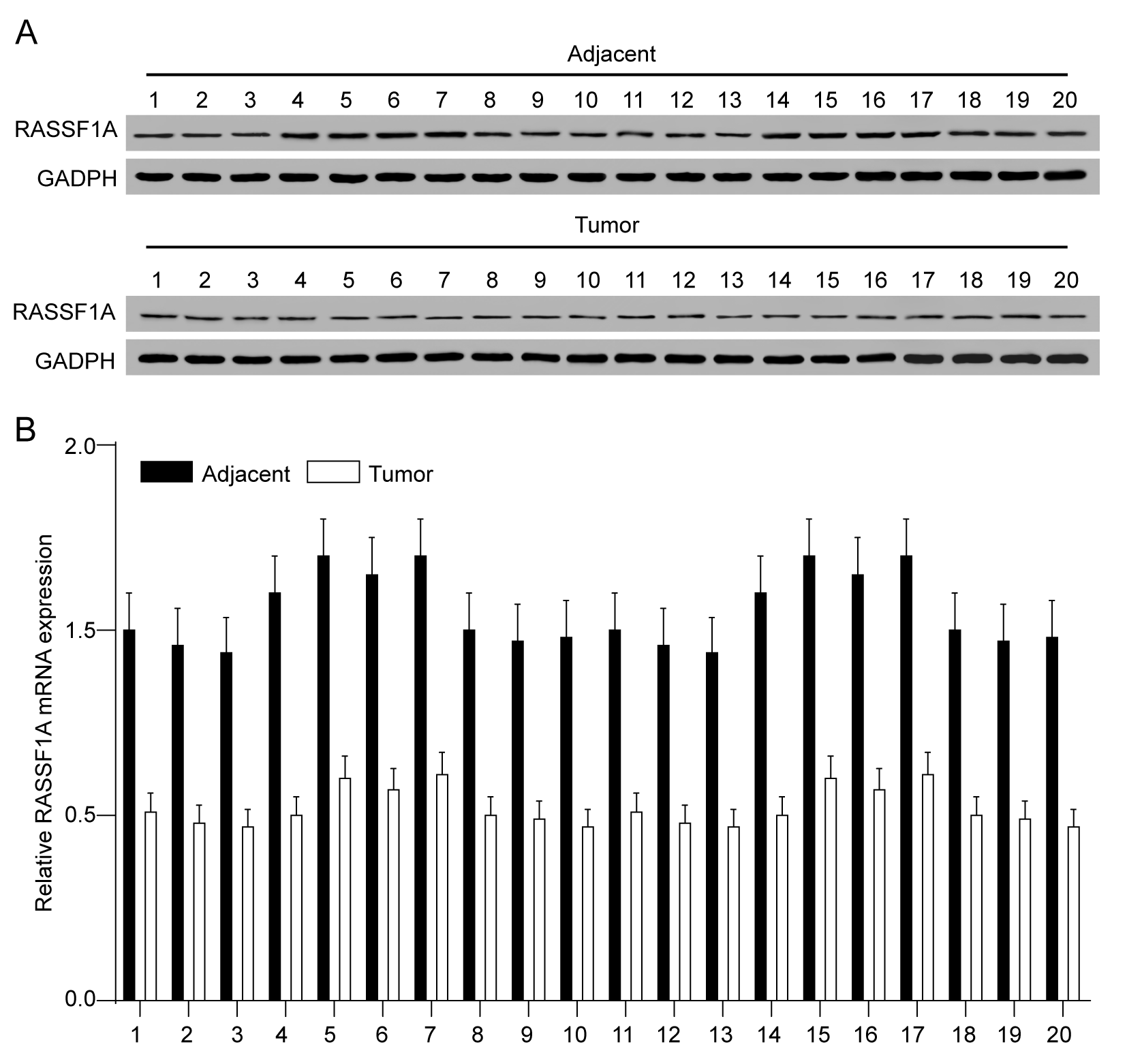

Supplement: Supplementary file 1 [file CPR-52-e12548-s001.tif]
